# A Monoallelic Deletion of the TcCRT Gene Increases the Attenuation of a Cultured *Trypanosoma cruzi* Strain, Protecting against an *In Vivo* Virulent Challenge

**DOI:** 10.1371/journal.pntd.0002696

**Published:** 2014-02-13

**Authors:** Fernando J. Sánchez-Valdéz, Cecilia Pérez Brandán, Galia Ramírez, Alejandro D. Uncos, M. Paola Zago, Rubén O. Cimino, Rubén M. Cardozo, Jorge D. Marco, Arturo Ferreira, Miguel Ángel Basombrío

**Affiliations:** 1 Instituto de Patología Experimental–CONICET, Universidad Nacional de Salta, Salta, Argentina; 2 Departamento de Medicina Preventiva Animal, Facultad de Ciencias Veterinarias y Pecuarias, Universidad de Chile, Santiago, Chile; 3 Cátedra de Química Biológica, Universidad Nacional de Salta, Salta, Argentina; 4 Programa Disciplinario de Inmunología, Instituto de Ciencias Biomédicas, Facultad de Medicina, Universidad de Chile, Santiago, Chile; US Food and Drug Administration, United States of America

## Abstract

*Trypanosoma cruzi* calreticulin (TcCRT) is a virulence factor that binds complement C1, thus inhibiting the activation of the classical complement pathway and generating pro-phagocytic signals that increase parasite infectivity. In a previous work, we characterized a clonal cell line lacking one TcCRT allele (TcCRT+/−) and another overexpressing it (TcCRT+), both derived from the attenuated TCC *T. cruzi* strain. The TcCRT+/− mutant was highly susceptible to killing by the complement machinery and presented a remarkable reduced propagation and differentiation rate both *in vitro* and *in vivo*. In this report, we have extended these studies to assess, in a mouse model of disease, the virulence, immunogenicity and safety of the mutant as an experimental vaccine. Balb/c mice were inoculated with TcCRT+/− parasites and followed-up during a 6-month period. Mutant parasites were not detected by sensitive techniques, even after mice immune suppression. Total anti-*T. cruzi* IgG levels were undetectable in TcCRT+/− inoculated mice and the genetic alteration was stable after long-term infection and it did not revert back to wild type form. Most importantly, immunization with TcCRT+/− parasites induces a highly protective response after challenge with a virulent *T. cruzi* strain, as evidenced by lower parasite density, mortality, spleen index and tissue inflammatory response. TcCRT+/− clones are restricted in two important properties conferred by TcCRT and indirectly by C1q: their ability to evade the host immune response and their virulence. Therefore, deletion of one copy of the TcCRT gene in the attenuated TCC strain generated a safe and irreversibly gene-deleted live attenuated parasite with high immunoprotective properties. Our results also contribute to endorse the important role of TcCRT as a *T. cruzi* virulence factor.

## Author Summary

*Trypanosoma cruzi* is a protozoan parasite which infects 9 million people in Latin America. Currently there is no vaccine to prevent this disease. Therefore, different approaches or alternatives are urgently needed to identify new protective immunogens. Live vaccines are likely to be most effective in inducing protection; however, safety issues associated with their use have been raised. Hence, we genetically manipulated an attenuated strain of *T. cruzi* as a safety device to rule out the possibility of reversion to the virulent phenotype. The genetically modified parasites were highly susceptible to killing by the complement machinery and presented a reduced propagation and differentiation rate. We have extended these studies to assess, the virulence, immunogenicity and safety of the mutant as an experimental vaccine. Accordingly, we show that genetically modified parasites present attenuated virulence in mice. The genetic alteration was stable and, after long term infection, it did not revert back to wild type form. Furthermore, after challenge with a virulent *T. cruzi* strain, mutant immunization induces a highly protective response evidenced by significantly lowered parasite density, mortality, spleen weight index and tissue inflammatory response. Our study provides new insights into the host-pathogen interactions and into the use and evaluation of irreversibly gene-deleted live attenuated parasites to protect against Chagas disease.

## Introduction

Chagas disease is a neglected tropical ailment caused by the flagellate protozoan *Trypanosoma cruzi*. It is estimated that 12–20 million people are infected worldwide causing 10–50,000 deaths/year [1]. Vector control strategies were not entirely successful mainly due to the inaccessibility and the vast distances that separate endemic areas. Transmission, despite the spraying of insecticides, has been increasing in parts of Argentina, Venezuela and Brazil [2]. In addition, the cases of Chagas disease have raised in many parts of South America and have spread globally because of immigration into non-endemic areas in developed countries [3], [4], [5]. Drugs used for treatment have serious adverse effects and do not cure the chronic stage [6]. However, vaccination to protect the 40–100 million individuals at risk of acquiring this serious disease has not been well developed or entered in human trials.

Considering *T. cruzi* complexity, with a genome of more than 12,000 genes and four distinct life stages, DNA and peptide vaccination for Chagas disease is insufficient and has, so far, not been reported to induce sterile immunity after challenge [7]. Currently, there is an increased interest in the development of irreversibly gene-deleted live attenuated parasites, as a possible mechanism to reduce the risk of reversion to virulence. There is considerable evidence in genetically modified organisms such as *Toxoplasma*, *Plasmodium* and *Leishmania*, which argues for the usefulness and effectiveness of these parasites as promising immunogens [8], [9], [10], [11], [12], [13], [14], [15], [16]. In *T. cruzi,* unfortunately, there are so far only five studies of vaccination using genetically attenuated strains [17], [18], [19], [20], [21]. The advantages of using this kind of immunogens are: (1) They can provide the full spectrum of relevant native epitopes and immune stimulating molecules, such as Toll-like receptors organized together, which would generate a high immunogenicity, unlike other types of vaccines that offer only a restricted spectrum of immunogens. (2) They can be manipulated to develop multiple genetic modifications. (3) They undergo antigen processing and presentation as in the case of virulent infection. (4) They generate, after inoculation, a strong and long lasting protective response compared with other experimental *T. cruzi* vaccines [7]. (5) They can be grown in axenic conditions with a lower economic production cost than other vaccine strategies [22].

The TCC wild type strain does not produce considerable tissue lesions or bloodstream parasite levels detectable by fresh blood mounts in rats [23]. Immunization with TCC provided, after a virulent challenge, a strong immune protection against virulent *T. cruzi* infections [24], [25], also evidenced when the challenge was performed using 17 isolates of *T. cruzi* obtained in an extensive endemic area of the Province of Salta, Argentina [26]. A strong control of parasitemia and tissue damage was observed in mice challenged a year after immunization [27], [28]. The protective effect of TCC was extended to field experiments in guinea pigs [25] and dogs [29]. Unfortunately, the TCC attenuation is genetically undefined and the possibility of reversion to the virulent phenotype cannot be excluded. In order to add a safety mechanism to prevent this reversion, in a previous work, we generated and characterized a TCC clonal cell line that lacks a TcCRT allele (TcCRT+/–) and another clone overexpressing it (TcCRT+). TcCRT is a *T. cruzi* virulence factor, that after being translocated from the endoplasmic reticulum (ER) to the area of flagellum emergence, can hijack the complement C1 component, inhibiting the activation of the classical and lectin complement pathways at their earliest stages [30], [31], [32] and producing pro-phagocytic signals increasing parasite infectivity [33]. Recently, an important role of TcCRT in the C1-dependent *T. cruzi* infectivity of human placenta explants has been determined in one of our laboratories, thus providing a plausible mechanism for congenital transmission of this infection [34]. In our previous work, we determined that the TcCRT+/– mutant contained about 6-fold less TcCRT polypeptide than wild type parasites [35]. Moreover, parasites overexpressing TcCRT contained about 2-fold more TcCRT polypeptide than wild type parasites. Consequently, monoallelic mutant parasites were significantly more susceptible to killing by the complement machinery. On the contrary, TcCRT+ parasites showed higher levels of resistance to killing by the classical and lectin but not by the alternative complement activation pathways. The involvement of surface TcCRT in depleting C1 was confirmed through restoration of serum killing activity by addition of exogenous C1. In axenic cultures, a reduced propagation rate of TcCRT+/– parasites was observed. Moreover, TcCRT+/– parasites presented a reduced rate of differentiation in *in vitro* and *in vivo* assays [35].

The previous studies led us to the objective of this report, to detect whether the TcCRT monoallelic deletion caused changes in the infectivity and immunoprotective behavior of the attenuated TCC strain.

## Materials and Methods

### Ethics statement

All animal protocols adhered to the National Institutes of Health (NIH) ‘‘Guide for the care and use of laboratory animals’’ and were approved by the Animal Ethics Committee of the School of Health Sciences, National University of Salta (N° 014-2011) [36].

### Trypanosomes cultures

A *T. cruzi* clone derived from the attenuated TCC strain [37], designated here as wild type, was used. Also, we used a clonal cell line lacking one TcCRT allele (TcCRT+/−) and a recombinant *T. cruzi* clone that overexpresses the TcCRT polypeptide (TcCRT+) [35]. Epimastigotes were grown at 28°C in liver infusion-tryptose medium (LIT) supplemented with 10% fetal bovine serum decomplemented at 56°C for 60 min., 20 µg hemin (Sigma, St. Louis, MO, USA),100 IU of penicillin and 100 µg/ml streptomycin. To obtain metacyclic trypomastigotes, epimastigote forms were allowed to differentiate by adding 10% w/v triatomine gut homogenate to the cultures [38]. The percentage of metacyclic forms was recorded daily in a Neubauer chamber. In addition, we used the Tulahuén strain and a highly infective isolate recently characterized [39].

### Hemocultures and PCR

Hemocultures were performed by seeding 200 µl of heparinized blood into 2 ml of LIT under sterile conditions; the cultures were incubated at 28°C and scanned for motile parasites under an inverted microscope on days 15, 30, 45, and 60. PCR for *T. cruzi* detection was also performed. Briefly, 700 µl of blood from each inoculated animal was processed. Kinetoplast DNA was amplified using primers 121 (5′-AAATAATGTACGGGTGAGATGCATGA-3′) and 122 (5′-GTTCGATTGGGGTTGGTGTAATATA-3′). Sample storage, DNA extraction, amplification, electrophoresis and staining were performed as previously described [40].

### Mutation stability

To assess the stability of the mutation, we used TcCRT+/– and TCC wild type parasites recovered from hemocultures performed on nude mice on day 90 post-infection (p.i.) after immunosuppression with cyclophosphamide. These parasites were grown and expanded in LIT medium. Genomic DNA was purified using the phenol–chloroform method. Diagnostic PCR analysis confirmed sequences corresponding to TcCRT and HYG gene. Primers used were: Pair 1, to amplify the entire TcCRT CDS (1.2 Kb), CRT1 (5'-GCCAGATATCATGAGGAGAAATGACATAAA-3') which anneals into the TcCRT initiation codon and CRT2 (5'-TCCTCTCGAGTCAAAACTTTCCCCACCGAA-3'), for the stop codon. Pair 2, to amplify the CDS of HYG gene (0.96 kb), H1 (5'-CGTCTGTCGAGAAGTTTCTG-3') which anneals into the HYG initiation codon and H2 (5'-GAAGTACTCGCCGATAGTG-3') for the stop codon. Pair 3, CRT 7 (5'-CCTTCCGATGGCATTAGC-3') which anneals upstream of TcCRT gene plus primer H4 (5'-CTCGCTCCAGTCAATGACC-3') for the HYG sequence (1.4 kb). Pair 4, CRT93 (5'-ATTCCAAACAACATTGCCGT-3') which anneals downstream of TcCRT gene plus H6 (5'-GGACCGATGGCTGTGTAGAAGTACTCGCCGATAGTGG-3') for the HYG sequence (1.4 kb).

### Serological determinations

Total Immunoglobulin G antibodies against *T. cruzi* were measured by Enzyme-linked Immunosorbent Assay (ELISA) using *T. cruzi* epimastigote homogenates (2 µg/well) as antigens. Dilutions of sera, anti-mouse IgG as a secondary antibody (Sigma, St. Louis, MO, USA) and conjugate were 1/100; 1/2,500 and 1/16,000 respectively. The antibody concentration was expressed as the optical density at 490-nm wavelength.

### *In vivo* infectivity assays

Male Balb/c inbred or athymic nude (nu/nu) immunodeficient mice (about 1 month old) were inoculated intra-peritoneally (i.p.) with 5×10^5^ metacyclic TCC TcCRT+/–; TcCRT+ and wild type trypomastigotes. Balb/c mice were subjected to PCR (15, 30, 90, 180 and 220 days p.i.), hemoculture (15, 30, 90 and 220 days p.i.) and serological determination of antibody levels (20, 47, 60, 90 and 165 days p.i.) as described above. Nude mice were examined by PCR and hemoculture on day 15, 30 and 90 p.i. To improve the detection of latent infections, the last sample of both, Balb/c and nude mice, were obtained after immunosuppressive treatment with cyclophosphamide. The immunosuppression regimen is based on 5, 250 mg/kg cyclophosphamide doses administered during 10 days. Samples were collected 10 days after the last dose.

### Immunization assays

To test whether mutant *T. cruzi* clones induced immunological protection, groups of 6 Balb/c mice, about 1 month old, were inoculated i.p. with 5×10^5^ metacyclic TCC TcCRT+/–; TcCRT+ and wild type trypomastigotes. A control group was inoculated with 100 µl of PBS (day 0). On day 15 a boost similar to the initial inoculation was administered. On day 30, antibody levels from immunized mice were determined and, on day 120, all groups were challenged with 10^4^ blood trypomastigotes of a highly virulent *T. cruzi* TcVI isolate, recently characterized [39]. Blood was drawn from the tail tip of mice, under slight ether anesthesia using heparinized, calibrated capillary tubes. Ten microliters of blood were placed between slide and cover slip and the number of parasites per 100 fields was recorded microscopically (40X) twice a week. Then, the number of parasites per 100 fields (parasitemia) was recorded from fresh blood mounts under microscope (40X). Finally, on day 60 post-challenge, surviving animals were sacrificed, spleen index and the presence of histological damage was measured in tissue samples.

### Histopathology

Tissue samples from heart and quadriceps muscle were fixed in 10% formaldehyde and processed using routine histological techniques. Serial histological hematoxylin-eosin-stained sections (3–5 µm thick) were studied. We searched for lymphocytic infiltrates in areas averaging 53 mm^2^ for heart and 38 mm^2^ for quadriceps muscle, scanning at least three sections per organ. Quantification of the inflammatory response was scored blindly as severe (+++: presence of foci containing numerous inflammatory cells covering at least half of the sections surface), moderate (++: large inflammatory foci covering up to ¼ of the section surface), slight (+: presence of small and isolated inflammatory foci) or absent (–: no presence of foci or inflammatory cells).

### Spleen index

Body and spleen weight were determined to calculate the spleen index (spleen index  =  spleen weight X 100/body weight) as an indirect effect of infection severity.

### Statistical analysis

The Mann-Whitney U tests and one-way variance analysis (ANOVA) of the GraphPad Prism version 5.0 software were used. Values are expressed as means ± standard error of mean of at least three separate experiments. P values equal to or minor that 0.05 were considered as significant.

## Results

### TcCRT+/– parasites showed attenuated virulence

To determine whether the TCC mutant parasites were capable of infecting and survive for long periods of time in the host, we monitored their *in vivo* infectivity and persistence. TCC TcCRT+/–; TcCRT+ and wild type epimastigotes were transformed into metacyclic trypomastigotes and inoculated (5×10^5^) in Balb/c and nude mice. Since the *T. cruzi* TCC strain is attenuated it is not possible to detect circulating parasites in blood samples by fresh blood mounts. Therefore, infection was detected by more sensitive methods (hemoculture and PCR). No positive hemocultures were obtained from any immunocompetent Balb/c mice inoculated with the three clones at any of the evaluated time points (Table 1). However**,** PCR determinations showed different infection patterns. No positive PCR was detected in TcCRT+/– immunocompetent inoculated mice throughout the follow-up, beyond 200 days, and even after immunosuppression. In contrast, positive reactions were found in all animals infected with wild type parasites. After immunosuppression, 2/3 of wild type inoculated mice were positive. All mice infected with TcCRT+ parasites presented a behavior similar to the wild type strain (Table 1). Thus, the attenuated TCC *T. cruzi* strain could be rendered even less virulent than wild type via the targeted deletion of one TcCRT allele.

**Table 1:** Infectivity and persistence of TcCRT mutants in Balb/c and nude mice.

		(Positive mice)/(total mice studied)								
		PCR					Hemoculture			
Mouse strain	*T. cruzi* clone	15	30	90	180	220	15	30	90	220
Balb/c	TcCRT+/–	0/5	0/5	0/5	0/4	0/3*	0/5	0/5	0/4	0/3*
	wild type	5/5	5/5	5/5	5/5	2/3*	0/5	0/5	ND	0/2*
	TcCRT+	4/5	5/5	4/4	4/4	2/2*	0/5	0/5	ND	0/2*
Nude	TcCRT+/–	3/4	4/4	0/1*	ND	ND	4/4	3/4	1/1*	ND
	wild type	5/6	3/4	1/1*	ND	ND	5/6	4/4	1/1*	ND
	TcCRT+	4/6	4/5	1/2*	ND	ND	4/6	4/5	0/2*	ND

Infectivity in mice infected with 5×10^5^ metacyclic TcCRT+/–, wild type and TcCRT+ trypomastigote clones in Balb/c and nude mice, as detected by hemoculture and PCR. ND =  not done. Numbers in the third line refer to days p.i. (*) samples obtained after cyclophosphamide immunosuppression.

Using nude mice we detected an increased rate of infection by PCR and hemoculture in all three experimental groups. No differences were found between the strains at any time p.i. As expected, after immunosuppression we detected a high mortality in all groups (Table 1).

### Infection with TcCRT+/– parasites induces lower levels of anti-*T. cruzi* antibodies

**Figure 1:**
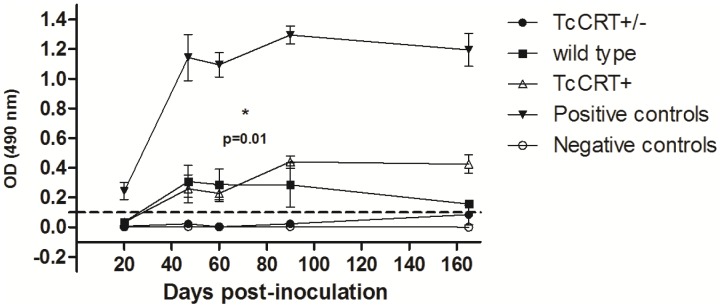
TcCRT+/– inoculated mice showed significant reduction of specific anti *T. cruzi* antibody levels. BALB/c mice were inoculated with 5×10^5^ metacyclic trypomastigotes. Results are expressed as the ratio of the absorbance of each serum sample at 490-nm. Dotted lines: Cut-off value adopted for positivity calculated as the mean of the values obtained for the negative controls (inoculated with PBS) plus three times the standard deviation. Serum samples were taken at the intervals indicated. TcCRT+/– inoculated mice showed significant reduction of antibody levels compared to those inoculated with wild type and TcCRT+ clones (p = 0.01 for both cases). Positive controls: serum from mice infected with the virulent Tulahuén *T. cruzi* strain.

We determined serum antibody levels in BALB/c mice infected with the three parasite clones on acute and chronic stages of infection and disease developmenRTt. TcC+/– infected mice showed undetectable antibody levels (p = 0.01) contrasting with mice inoculated with both wild type and TcCRT+ parasites. In fact, the TcCRT+/– values were comparable to those obtained from the PBS-inoculated, negative controls. No differences between TcCRT+ and wild type were found (p = 0.84). As previously described [27], mice inoculated with the Tulahuén virulent strain (positive control) showed about six-fold higher antibody levels than those obtained with any of the TCC strains (Fig. 1).

### The TcCRT+/– mutation remains stable during chronic infection in mice

**Figure 2:**
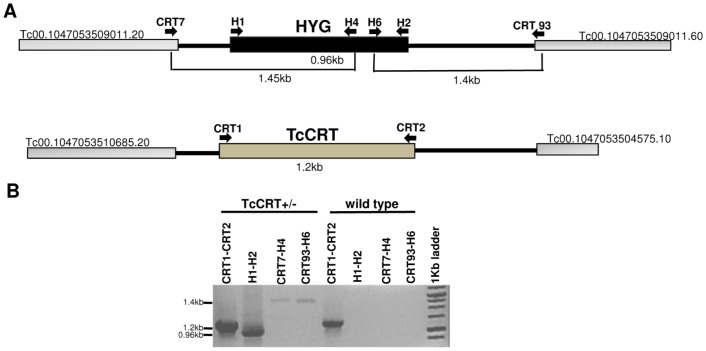
TcCRT+/– mutant parasites are stable after prolonged infection in mice. (A) Schematic representation of the TcCRT genomic locus in TcCRT+/– parasites (B) PCR analysis carried out from genomic DNA of TcCRT+/– and wild type parasites recovered from hemocultures of chronically infected mice. Primers: Pair CRT1-CRT2 and H1-H2 amplify the TcCRT (1.2 KB) and HYG CDS (0.96 kb), respectively. Pair CRT7-H4 amplifies the 5' UTR of TcCRT together with a fragment of the HYG CDS (1.45 kb). Pair CRT93-H6 amplifies the 3' UTR of TcCRT together with a fragment of the HYG CDS (1.4 kb). The sizes of the fragments correspond to those predicted for the replacement of TcCRT by the HYG gene.

To exclude the possibilities of cross-contamination, reversion of the genetic mutation or TcCRT locus instability, we determined whether the parasites isolated from hemocultures after long term infection in mice corresponded to mutant parasites. Genomic DNA was extracted from TcCRT+/– and wild type parasites grown on hemocultures at day 90 p.i. (Table 1). We amplified sequences corresponding to the TcCRT coding sequence (CDS) and the hygromycin phosphotransferase (HYG) marker gene. The sizes of amplified fragments in the DNA of the recovered parasites corresponded to those predicted for the replacement of TcCRT by the HYG gene (Fig. 2).

Additionally, the antibiotic resistance of TcCRT+/– and wild type parasites were tested. Only TcCRT+/– parasites survived in the presence of 300 µg/ml Hygromicin B mediated by the HYG resistance gene at the deleted TcCRT allele (data not shown). Thus, this evidence showed that TcCRT+/– parasites conserved the targeted allele introduced by homologous recombination, that there is no cross-contamination and that the locus remained stable throughout the infection cycle in the mammalian host.

### Infection with TcCRT+/– parasites induces protective immunity

**Figure 3:**
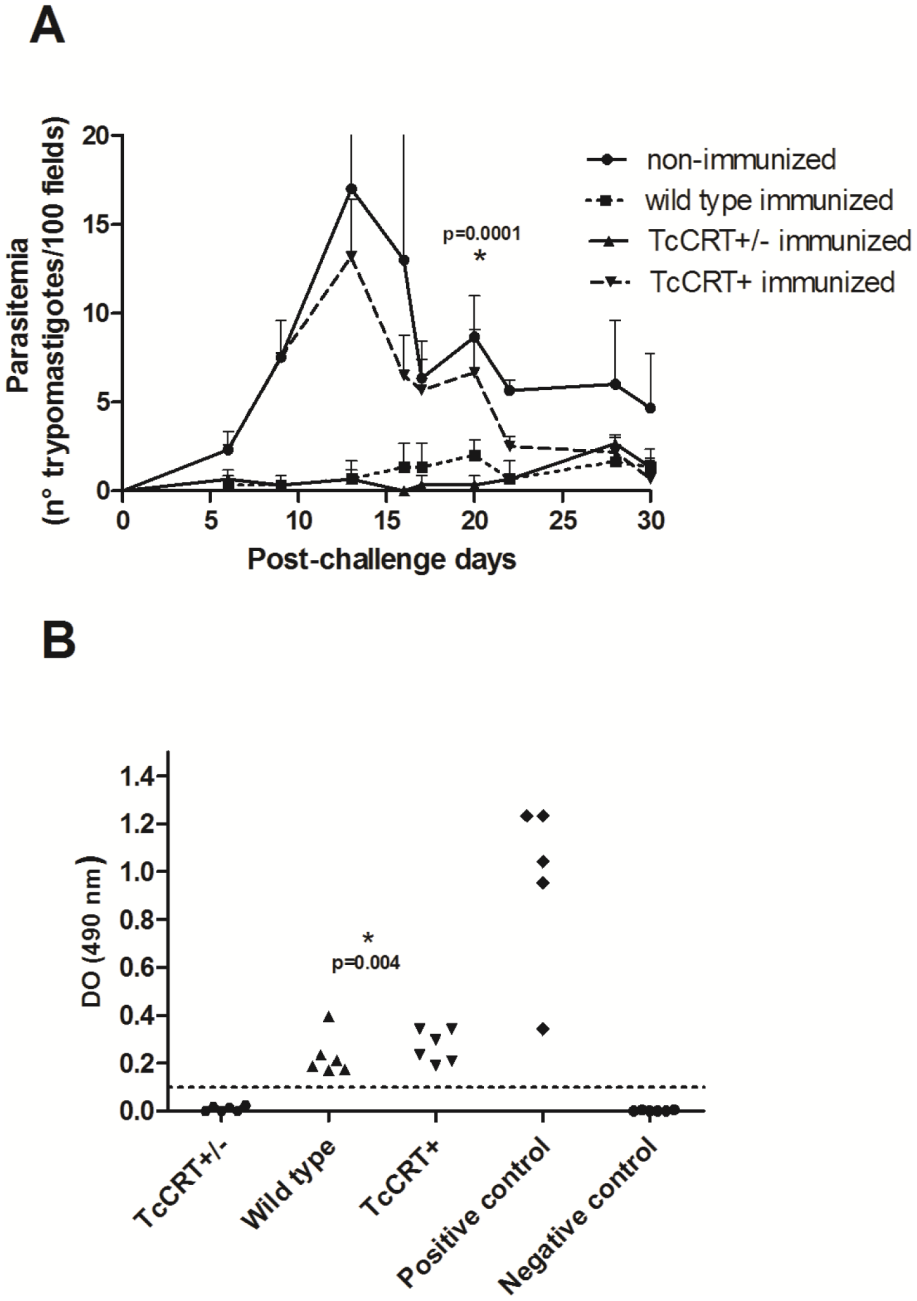
Inoculation of TcCRT+/– mutant parasites protects mice against a virulent *T. cruzi* challenge. (A) Balb/c mice were primed and boosted with metacyclic trypomastigotes TCC wild type, TcCRT+, TcCRT+/– or PBS. On day 120, all mice were challenged with 10^4^ bloodstream forms of a virulent *T. cruzi* TcVI isolate. Note the protection (p = 0.0001) in TcCRT+/– and wild type-preinoculated mice. (B) Dispersion diagrams of specific anti-*T. cruzi* antibody levels elicited in either naive mice (non- immunized) and those immunized and boosted with 5×10^5^ metacyclic trypomastigotes TcCRT+/–, wild type or TcCRT+ clone. The results are expressed as the ratio of the absorbance of each serum sample at 490-nm. Dotted lines: Cut-off value adopted for positivity calculated as the mean of values obtained for the negative controls plus three standard deviations. Serum samples were taken at day 30 post-priming. TcCRT+/– immunized mice showed undetectable antibody levels as compared to mice inoculated with wild type and TcCRT+ clones (p = 0.006 for both cases). Data (mean ± SD) presented are representative of three independent experiments (n = 6/group/experiment).

To assess the immunoprotective capacity of mutant parasites against a subsequent reinfection with virulent parasites, groups of six BALB/c mice were inoculated with 5×10^5^ metacyclic trypomastigotes of each of the three clones plus a naive, sham-preinoculated control group. At day 15 a booster similar to the initial inoculation was administered. To determine whether this immunization regimen induces an immune response, blood samples were taken during the immunization phase on day 30 post-priming. After 120 days, these mice together with controls were challenged with 10,000 bloodstream trypomastigotes of a virulent *T. cruzi* isolate [39]. The protective response generated by immunizing with TcCRT+/– and wild type was significantly higher than in the non-immunized group (p = 0.0001). Mice immunized with TcCRT+/– and wild type parasites showed, after challenge, reduced levels of circulating parasites in peripheral blood, ranging between 0–3 parasites per 100 microscopic fields throughout follow-up, demonstrating the protection afforded by immunization. Parasitemia curves between wild type and TcCRT+/– immunized groups are not significantly different (p  = 0.22). These results showed that deletion of a TcCRT allele does not modify the protective response induced by TCC wild type parasites. In contrast, mice immunized with TcCRT+ did not afford protection (Fig 3A). Non-immunized control mice presented high parasitemia with peaks between days 13 and 16, at a time when there was 50% mortality. As expected, these mice showed high parasitemia before death, thus explaining the wide dispersion of the data at that time (Fig 3A). In contrast, in the remaining experimental groups no mortality was recorded. TcCRT+/– immunized and boosted mice showed undetectable specific anti-*T. cruzi* antibody levels (similar to those obtained in the non-immunized, negative controls) compared to the levels found in both wild type and TcCRT+ (p = 0.004) and clearly different from those obtained from mice infected with Tulahuén parasites (Fig 3B).

**Figure 4:**
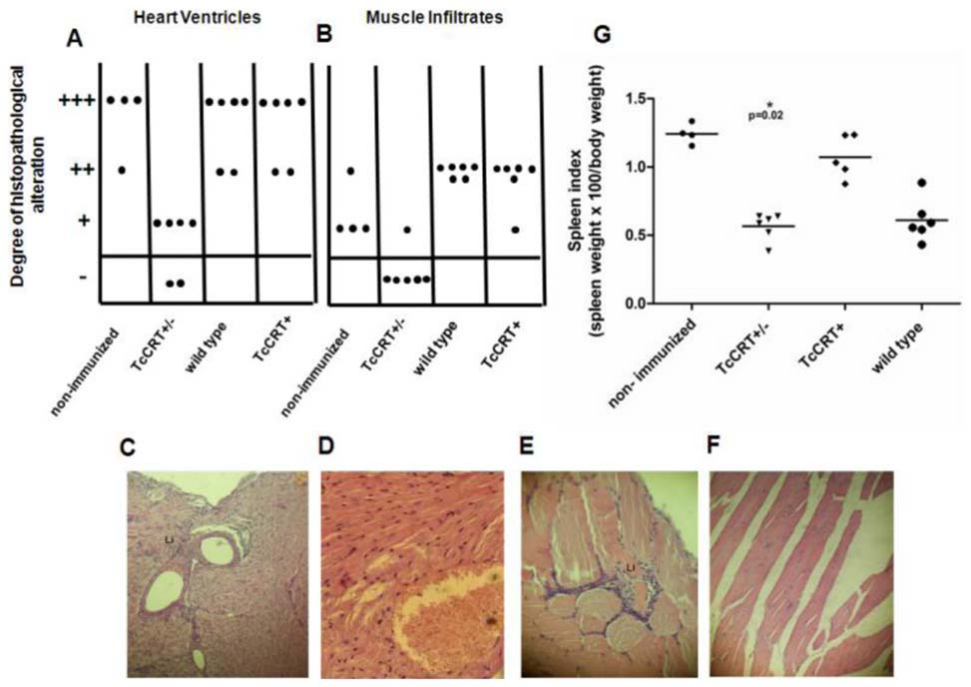
TcCRT+/– immunization decreases tissue inflammatory response and spleen indexes in challenged mice. Autopsies were performed on mice 4 months post-priming and 2 months after virulent challenge. Dispersion diagrams of histopathological alterations in hearth muscle (A) and skeletal tissue (B). The inflammatory responses were graded as absent (–), slight (+), moderate (++), and severe (+++). Each dot represents a mouse. Representative images of H&E staining (blue: nuclear, pink: muscle/cytoplasm/keratin) of heart tissue (C and D) and skeletal muscle sections (E and F) from non-immunized and TcCRT+/– mice respectively (magnification, 25X). Li, Lymphocytic infiltrates. (G) Spleen indexes on day 60 post-challenge. Mice inoculated with TcCRT+/– parasites present lower spleen indexes compared with non-immunized controls (p = 0.02) and TcCRT+ parasites (p = 0.004).

Autopsies were performed on mice 4 months after priming with TCC TcCRT+/–; TcCRT+ or wild type trypomastigotes and 2 months after a virulent *T. cruzi* challenge. Non-immunized, wild type and TcCRT+ mice presented severe inflammatory response throughout the heart tissue, however, this response was extensively reduced in TcCRT+/– immunized mice (p = 0.002) (Fig. 4A), thus confirming the protective effect conferred by previous immunization with these parasites. The same effect was observed in muscle tissue: non immunized, wild type and TcCRT+ mice presented moderate to slight cellular damage that was reduced in TcCRT+/– immunized mice (p = 0.0007) (Fig. 4B).

Splenomegaly is a macroscopic manifestation of the expansion of B- and T-lymphoid cell populations produced by the infection of mice with *T. cruzi* [41]. Thus, the spleen index represents an indirect effect of infection severity. Spleen index at day 60 post-challenge was significantly decreased in TcCRT+/– and wild type immunized mice compared to that in the non-immunized controls (p = 0.02 for both cases). However, TcCRT+ immunized mice presented no differences (p = 0.14) with non-immunized controls (Fig. 4G).

## Discussion

In a previous work, we have characterized a mutant cell line that lacks a TcCRT allele (TcCRT+/–), with bases on the attenuated TCC *T. cruzi* strain. We showed that TcCRT+/– epimastigotes contained about 6-fold less TcCRT polypeptide than wild type parasites. Moreover, they were significantly susceptible to killing by the complement machinery and presented a reduced *in vitro* propagation and differentiation rate. In addition, we generated another clonal cell line that over-expresses TcCRT (TcCRT+) and showed high resistance levels to complement attack [35]. Furthermore, it was not possible to generate biallelic TcCRT–/– null mutant clones, perhaps a reflection of the essential character of the TcCRT protein for parasite survival.

TCC wild type infection is hardly detected in immunocompetent animal models due to the attenuation of this strain. The use of highly sensitive methods such as immunosupression regimens followed by PCR and hemoculture is usually required. When inoculated in Balb/c mice, and during a 6-month follow-up period, mutant TcCRT+/– parasites were not detected by either of these techniques, even after immunosupression (Table 1). TcCRT is highly immunogenic in different animal species [42]. Most humans infected with *T. cruzi* possess anti-TcCRT antibodies [43]. However, levels of specific antibodies in TcCRT+/– inoculated mice were even more reduced as compared to mice inoculated with wild type or TcCRT+ and, as described [27], with the highly infective Tulahuén strain (Fig. 1).

The increased virulence attenuation of TcCRT+/– in mice could probably be related to increased complement susceptibility and to the deposition of C1q on the parasite surface, configuring a strategy called "apoptotic mimicry". In infective trypomastigotes, TcCRT is translocated from the ER to the area of flagellum emergence where it could hijack C1q resulting in an increased affinity for host cells [32], [33], [44]. Previous reports affirm that the C1q binding on the *T. cruzi* trypomastigote surface increases parasite infectivity [45] and thus, any disruption of TcCRT/C1q interaction may result in a reduction of infectivity both, *in vitro* and *in vivo* [33], [34]. Furthermore, apoptotic mammalian cells express surface ligands with high C1q affinities, among them, the calreticulin orthologue. C1q-coating over apoptotic cells produces pro-phagocytic “eat me” signals that promote clearance of apoptotic bodies conducted by phagocytic cells [46], [47]. One of our laboratories [33], proposed that *T. cruzi* expressing TcCRT mimic the “eat me” signals, promoting C1q coating, phagocytic cell chemotaxis and increasing parasite infectivity in the early stages of infection. In our work, the TcCRT allele deletion and synthesis reduction [35], possibly generated a lower capacity to capture C1 thereby inducing lower pro-phagocytic signals and reduced infectivity of phagocytic cells in the early stages of infection. In a negative feedback, the limited invasion of phagocityc cells would help TcCRT+/– parasites to stay free, for a longer period of time, and exposed to the complement lytic action in the bloodstream system of the host. These properties may have contributed to the important TcCRT+/– infectivity attenuation (Table 1).

In addition, antibodies aggregated to the *T. cruzi* surface antigens (including the anti-TcCRT antibodies) through their Fc regions have a high affinity for C1q [33]. Thus the apparent paradox that C1q-fixing antibodies, rather than preventing parasite replication, contribute to increase their infectivity, is explained. Thus, pretreatment with anti-TcCRT (Fab')_2_ fragments (which lack the Fc fragment of C1q binding) produces the disruption of TcCRT/C1q with serious negative impact on the *in vivo* e *in vitro* infectivity [33]. As expected, the TcCRT+/– attenuated line did not produce detectable specific anti-*T. cruzi* antibodies (Fig.1) probably causing a limited C1q deposit on the parasite surface which, in turn, would contribute to diminish phagocytic signals and hence parasite infectivity. In contrast, mice inoculated with TcCRT+ and wild type showed an increased level in antibody titers compared to TcCRT+/–, which would generate a denser C1q coating. This phenomenon may explain the divergences in infectivity of TcCRT+/–, TcCRT+ and wild type parasites.

We were unable to recover infecting parasites from immunocompetent mice by hemoculture. However, we could detect parasite DNA by PCR in those mice infected with TCC wild type and TcCRT+ parasites (Table 1). This is probably a consequence of both a lower density of circulating parasites and a greater PCR sensitivity for detection of *T. cruzi* in mouse blood (X 20) as compared with hemoculture [27].

In agreement with our hypothesis, mice inoculated with TcCRT+ and wild type parasites infected a high percentage of mice, although without detectable differences between these groups.

It is unclear whether TcCRT+/– parasites did infect. However, the possibility that infection occurs is favored by the fact that an adaptive protective status was verified when the animals were challenged 4 months after a primary infection. Since only marginal antibody levels were occasionally detected, protection maybe cellular rather than humoral, issues now under investigation in our laboratories.

Using immunedeficient nu/nu mice, infections caused by the three parasite populations could be detected in a high proportion of mice and even in hemocultures (Table 1). These results confirm previous studies from our laboratory, showing that the TCC wild type strain infects immature or immunocompromised animals [24], [48]. These results suggest that although TcCRT+/– infectivity is attenuated, the suppression of host immunity allows the replication and persistence of these parasites in animals. A similar behavior was observed in the *dhfr-ts* (dihydrofolate reductase-thymidylate synthase) single mutant, also developed on the TCC *T. cruzi* strain. This mutant showed a reduced infectivity in immunocompetent mice and as in this work, no mutant parasites could be recovered from hemocultures [20].

The virulence reduction in genetically modified parasites in mice models has previously been reported for genes Tc52 [49] and oligopeptidase B [50]. In our laboratory, this phenomenon was observed working with mutant gp72 genes [19], cub (calmodulin-ubiquitin) [17], lyt1 [18] and *dhfr-ts* [20].

We have extensively studied the TCC *T. cruzi* strain as a live attenuated experimental vaccine [28], [29], [51]. The molecular basis of the TCC attenuation is unknown. Thus, we incorporated a rational attenuation mechanism (targeted gene deletion) as a safety device to eliminate the possibility of reversion to a virulent phenotype. In this regard, we tried to rule out the possible reversion of the TcCRT+/– genetic modifications during the chronic stage of the disease in mice. We recovered TcCRT+/– parasites from nude mice at day 90 p.i. (Table 1) and detected sequences corresponding to the TcCRT locus engineering. Thus, the TcCRT+/– mutation is genetically stable in chronically infected mice and there is no reversion to the TCC wild type genotype (Fig.2). Furthermore, the same experiment ruled out strain cross contamination during handling in the laboratory.

Moreover, we tested whether the TcCRT+/– attenuation affects the protective capacity of the TCC wild type parasites against a virulent challenge. Our results suggest that the deletion of one TcCRT allele did not change the already reported immunoprotection induced by TCC wild type parasites [28]. Using TcCRT+/– immunized mice we did not obtain, after a virulent challenge, a sterilizing protective response, although, we achieved low parasite density, mortality (Fig.3A) and a significantly reduced tissue inflammatory response (Fig.4A–F) and spleen index (Fig.4G). Infection with the parental TCC clone has been shown to be protective, in spite of the fact that it generates inflammatory foci in cardiac tissue [23]. When we inactivated one of the TcCRT alleles, a significant decrease in local inflammation is recorded, perhaps a reflection of an impaired virulence. Certainly, the most important fact was that the protective response was achieved at the cost of a possible primary infection with attenuated TcCRT+/– parasites which could not be detected by our most sensitive methods during a six month follow-up and even after immunosuppression of the infected mice (Table 1). It is crucial for vaccinating parasites not to persist in the organism and to discontinue the transmission cycle in peridomestic animals from endemic areas. This could impact over the Chagaś disease infection incidence.

In a previous work [33], mice immunization with TcCRT induced the generation of specific anti-TcCRT antibodies resulting in increased parasitemia of the *T. cruzi*-challenged mice. Most likely, as mentioned above, immunization with TcCRT induces C1q binding anti-TcCRT antibodies thus increasing the parasite infectivity in the challenged animals. According to this hypothesis, TcCRT+ immunized mice showed higher levels of specific anti-*T. cruzi* antibodies (Fig 3B) inducing an elevated parasitemia after challenge (Fig 3A). On the contrary, the TcCRT+/– attenuated line did not produce detectable antibodies (Fig 3B) or parasitemia post-challenge (Fig 3A).

Wild type TCC parasites are not detectable by direct blood examination, however, they could be detected by PCR in cyclophosphamide treated chronically infected mice (Table 1) or after hemoculture recovery. In this regard, the attenuated biological behavior of the TcCRT+/– mutants is interesting because if employed as live immunogens, an eventual (natural or induced) immunosuppression of the host should not produce the reactivation of the vaccinating parasites.

Inoculation and eventual infection with TcCRT+/– parasites did not induce detectable antibodies levels (Fig.1 and 3B). However, protection from *T. cruzi* infection is considered at present to be mediated primarily by cytotoxic T cells [52] and not by antibodies.

In summary, our results show that TcCRT+/– clones were restricted in two important properties conferred by TcCRT and indirectly by C1q: the ability to evade the host immune response, and their virulence status. Therefore, deletion of one copy of the TcCRT gene in the attenuated TCC strain resulted in the generation of a safe and irreversibly gene-deleted live attenuated parasite with high experimental immunoprotective properties.
